# Mimicking a natural pathway for *de novo* biosynthesis: natural vanillin production from accessible carbon sources

**DOI:** 10.1038/srep13670

**Published:** 2015-09-02

**Authors:** Jun Ni, Fei Tao, Huaiqing Du, Ping Xu

**Affiliations:** 1 State Key Laboratory of Microbial Metabolism, and School of Life Sciences & Biotechnology, Shanghai Jiao Tong University, Shanghai 200240, People’s Republic of China

## Abstract

Plant secondary metabolites have been attracting people’s attention for centuries, due to their potentials; however, their production is still difficult and costly. The rich diversity of microbes and microbial genome sequence data provide unprecedented gene resources that enable to develop efficient artificial pathways in microorganisms. Here, by mimicking a natural pathway of plants using microbial genes, a new metabolic route was developed in *E. coli* for the synthesis of vanillin, the most widely used flavoring agent. A series of factors were systematically investigated for raising production, including efficiency and suitability of genes, gene dosage, and culture media. The metabolically engineered strain produced 97.2 mg/L vanillin from l-tyrosine, 19.3 mg/L from glucose, 13.3 mg/L from xylose and 24.7 mg/L from glycerol. These results show that the metabolic route enables production of natural vanillin from low-cost substrates, suggesting that it is a good strategy to mimick natural pathways for artificial pathway design.

Vanillin (4-hydroxy-3-methoxybenzaldehyde) is one of the most widely used flavoring agents in the world. With an intensely and tenacious creamy vanilla-like odor, it is often used in foods, perfumes, beverages, and pharmaceuticals^1^. As a plant secondary metabolite, natural vanillin is extracted from the seedpods of orchids (*Vanilla planifolia*, *Vanilla tahitensis*, and *Vanilla pompona*). The annual worldwide consumption of vanillin exceeds 16,000 tons; however, owing to the slow growth of orchids and the low concentrations of vanillin in these plants (about 2% of the dry weight of cured vanilla beans), only about 0.25% of consumed vanillin originates from vanilla pods^2^. Most market demand is met by chemical synthesis of vanillin from lignin or fossil hydrocarbons, but the process is not environmentally friendly and it lacks substrate selectivity. Another drawback of chemical synthesis is that the synthetic, “unnatural” vanillin is sold for only $12 per kilogram, which is only 0.3% of the price of natural vanillin^3^. An alternative is the use of microbial biotechnology to produce vanillin from natural substrates; the resulting product is classified as natural vanillin under European and US food legislation^4^. Rising demand for natural products has led to the development of various biotechnological approaches for the production of vanillin. Phytochemicals such as ferulic acid are the major substrates used in natural vanillin production methods. Although different microorganisms capable of converting ferulic acid to vanillin have been isolated and studied in the past decade^5^,^6^,^7^, the high price of ferulic acid has limited its application.

Biosynthesis of vanillin from simple carbon sources like glucose is much more attractive because these sources are much cheaper and more readily available (glucose costs less than 30 cents per kilogram)^8^. Two artificial pathways have been developed for the production of vanillin from simple carbon sources. As shown in Supplementary Fig. 1, Frost *et al.* designed a pathway in recombinant *Escherichia coli* for the *de novo* biosynthesis of vanillic acid from glucose via the shikimic acid pathway; the vanillic acid was then enzymatically reduced to vanillin by aryl aldehyde dehydrogenase *in vitro*^9^. This route requires isolated dehydrogenase and costly cofactors, and only trace amounts of vanillin can be detected. Hansen *et al.* demonstrated *de novo* biosynthesis of vanillin from glucose in *Schizosaccharomyces pombe* and *Saccharomyces cerevisiae* by a similar route, but they introduced an aromatic carboxylic acid reductase gene to avoid the extracellular reaction (Supplementary Fig. 1). In *S. pombe* and *S. cerevisiae*, vanillin production was 65 and 45 mg/L, respectively^10^. Brochado *et al.* expressed a glycosyltransferase in the vanillin-producing *S. cerevisiae* strain to reduce product toxicity, and compared with the previous work, vanillin production was increased approximately fivefold in batch mode through *in silico* design^11^. However, the glycosylation step may have reduced the maximum theoretical yield^12^, and the use of dehydroshikimic acid may limit the aromatic amino acid biosynthesis pathway. Moreover, many alcohol dehydrogenases in yeast can act on vanillin and the precursor protocatechuic aldehyde, leading to loss of product. Recently, Kunjapur *et al.* used an *E. coli* with reduced aromatic aldehyde reduction as a platform for aromatic aldehyde biosynthesis. After the introduction of a pathway for the biosynthesis of vanillin, 119 ± 3 mg/L vanillin can be produced from glucose^13^.

The natural pathway for vanillin production that has evolved in plants could be much more efficient^14^,^15^. Mimicking and assembling the natural pathway in *E. coli* to synthesize vanillin from glucose may overcome the shortcomings of previous works. Based on research into construction of the phenylpropanoid pathway in microorganisms^16^,^17^,^18^,^19^, a simulated natural pathway including five enzymes was introduced into *E. coli* to synthesize vanillin from simple carbon sources. Compared with previous pathways, this new metabolic route converted a greater variety of substrates to vanillin, including l-tyrosine and glucose. Xylose (an important pentose component of renewable biomass feedstocks) and glycerol (an inexpensive byproduct of the biodiesel industry) were also used for the production of vanillin. To optimize vanillin production, we investigated the efficiency and suitability of biosynthetic genes from different sources, gene dosage effects, and different culture media.

## Results

### Pathway design and construction

**Figure 1:**
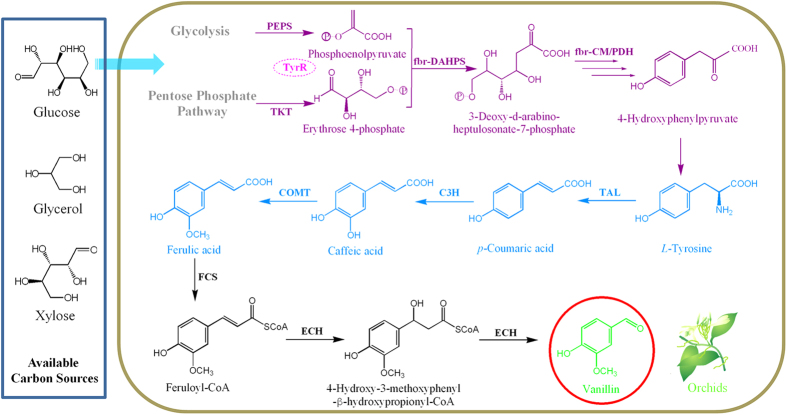
Artificial vanillin biosynthetic pathway constructed in *Escherichia coli* cells. The purple part shows the reconstruction of the common aromatic pathway for l-tyrosine overproduction; the global regulatory protein TyrR has been inactivated and four enzymes are overexpressed. As shown in the pale blue part, three enzymes are involved in the artificial pathway from tyrosine to ferulic acid. Black part in the right box shows the three steps from ferulic acid to vanillin, which are catalyzed by two enzymes. TyrR, a regulatory protein; PEPS, phosphoenolpyruvate synthetase; TKT, transketolase; fbr-DAHPS, fbr-3-deoxy-d-arabino-heptulosonate-7-phosphate synthase; fbr-CM/PDH, fbr-chorismate mutase/prephenate dehydrogenase; TAL, tyrosine ammonia-lyase; C3H, 4-coumarate 3-hydroxylase; COMT, caffeate *O*-methyltransferase; FCS, trans-feruloyl-CoA synthetase; ECH, enoyl-CoA hydratase/aldolase. The orchid was drawn by Jun Ni.

**Figure 2:**
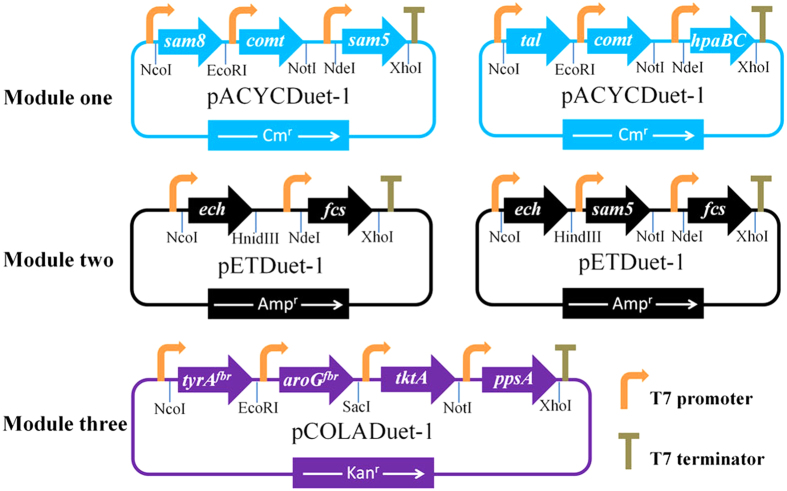
Schematic representation of artificial gene clusters used for vanillin production. A T7 promoter and ribosomal binding site precede each gene, and a T7 terminator is located downstream of each gene cluster. Module one contains the biosynthetic gene cluster used for ferulic acid production. Module two contains the gene cluster used for production of vanillin from ferulic acid. Module three was used for l-tyrosine overproduction. Cm^r^, chloromycetin resistance; Amp^r^, ampicillin resistance; Kan^r^, kanamycin resistance.

We designed the artificial vanillin biosynthetic pathway shown in Fig. 1. With five enzymes, including tyrosine ammonia-lyase (TAL; EC 4.3.1.23), 4-coumarate 3-hydroxylase (C3H; EC 1.14.14.9), caffeate *O*-methyltransferase (COMT; EC 2.1.1.68), trans-feruloyl-CoA synthetase (FCS; EC 6.2.1.34), and enoyl-CoA hydratase/aldolase (ECH; EC 4.2.1.17), l-tyrosine could be converted to vanillin. Grafting this artificial pathway into a tyrosine-overproducing *E. coli* strain would enable production of vanillin from simple carbon sources. To accomplish this, we constructed a single-knockout *E. coli* strain harboring three plasmids (Fig. 2).

Tyrosine ammonia-lyase (TAL) catalyzes non-oxidative deamination of l-tyrosine to 4-coumaric acid, and many microbial TALs and their corresponding genes have been reported in recent years^20^, including the *sam8* gene from the actinomycete *Saccharothrix espanaensis*^21^ and the *RsTAL* gene from the photosynthetic bacteriuma *Rhodobacter sphaeroides*^22^. 4-Coumarate 3-hydroxylase is a plant-specific cytochrome P450-dependent monooxygenase that converts 4-coumaric acid to caffeic acid by hydroxylation at the 3-position of the benzene ring. Because of the membrane-bound property and the instability of C3H, functional expression of this protein in bacterial systems is very challenging^23^. Recently, two microbial C3Hs from *S. espanaensis* and *E. coli* (encoded by *sam5* and *hpaBC*, respectively) were reported and their functions were characterized^21^,^24^. Caffeate *O*-methyltransferase can convert caffeic acid to ferulic acid^25^, and the *comt* gene from *Arabidopsis thaliana* was codon-optimized and synthesized for this purpose.

The vanillin synthase from *Vanilla planifolia* can directly convert ferulic acid to vanillin^15^; however, we failed to express it in *E. coli*. Instead, the *fcs* and *ech* genes (encoding FCS and ECH, respectively) were recently isolated from *Streptomyces* sp. strain V-1 and their functions were characterized in *E. coli*^26^. For conversion of ferulic acid to vanillin, we constructed a plasmid, *fcs-ech*/pET, bearing both of these genes. The next step was to introduce our artificial biosynthetic pathway into a tyrosine-overproducing strain. An *E. coli* strain carrying the plasmid *tyrA*^*fbr*^*-aroG*^*fbr*^*-tktA-ppsA*/pCOLA and a knockout mutation in *tyrR* fit our requirements^27^. Inactivation of TyrR-mediated regulation improves the phenotype of aromatic amino acid producers, which leads to an overflow of l-tyrosine biosynthesis. The plasmid expresses phosphoenolpyruvate synthetase (PEPS: *ppsA*), transketolase (TKT: *tktA*), feedback-inhibition-resistant (fbr) 3-deoxy-d-arabino-heptulosonate-7-phosphate synthase (fbr-DAHPS: *aroG*^*fbr*^), and fbr-chorismate mutase/prephenate dehydrogenase (fbr-CM/PDH: *tyrA*^*fbr*^). Higher expression of PEPS and TKT can increase carbon flow into the aromatic amino acid biosynthesis pathway, and fbr-DAHPS and fbr-CM/PDH can depress inhibition by metabolites.

Thus, our artificial vanillin biosynthetic system, inspired by naturally occurring biosynthetic pathways, comprises an *E. coli* strain with a knockout mutation, four overexpressed genes, and five exogenous genes. With this system, many simple carbon sources, such as l-tyrosine, glucose, xylose, and glycerol, may easily convert into vanillin.

### Comparison of two recombinant plasmids for ferulic acid production

Previous studies have found that some enzymes involved in the phenylpropanoid pathway are ineffective in recombinant *E. coli*^17^,^28^. Therefore, we compared key biosynthetic genes from different sources to obtain high-yield production of ferulic acid. To evaluate the performance of the genes from gram-positive bacteria (*sam8* and *sam5*) and gram-negative bacteria (*RsTAL* and *hpaBC*), *sam8*/pACYC and *tal*/pACYC were constructed first, and the production of 4-coumaric acid from l-tyrosine by *E. coli* carrying these plasmids was investigated. In a previous study, a large amount of insoluble TAL was found when a recombinant strain was cultured at 37 °C^17^; therefore, we cultured the transformed *E. coli* at 26 °C to reduce the formation of inclusion bodies. The recombinant strains were cultured in Luria-Bertani (LB) medium supplemented with an additional 2 g/L l-tyrosine and 0.2 mM IPTG. After 48 h, high-performance liquid chromatography (HPLC) and liquid chromatography–mass spectrometry (LC–MS) analysis showed that the concentration of 4-coumaric acid reached 875 mg/L and 821 mg/L in cultures of the recombinant strains containing *sam8*/pACYC and *tal*/pACYC, respectively (Table 1). There was no significant difference in 4-coumaric acid production between the two recombinant strains. Therefore, to future study the 4-coumaric acid conversion capacity of recombinant strains with genes from different sources, we introduced the *sam5* gene into *sam8*/pACYC and the *hpaBC* gene into *tal*/pACYC. The recombinant strains carrying *sam8*-*sam5*/pACYC and *tal*-*hpaBC*/pACYC were cultured in the same medium and under the same conditions described above, and the production of caffeic acid reached 136 mg/L and 44 mg/L, respectively, after 48 h of culture (Table 1). Because actinomycete genes might contain rarely used codons and alterations of mRNA structural elements they are often difficult to express in *E. coli*^29^. Nevertheless, the strain with *sam8-sam5*/pACYC produced 209% more caffeic acid than the strain with *tal-hpaBC*/pACYC. This may be caused by a side effect of the enzyme encoded by *hpaBC*, which can catalyze the conversion of tyrosine to l-DOPA and reduce the production of 4-coumaric acid^30^.

**Figure 3:**
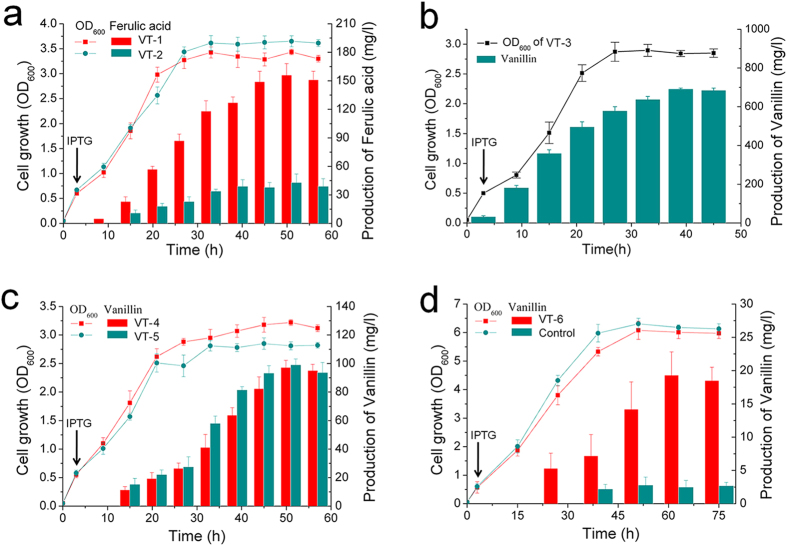
Fermentative production of ferulic acid and vanillin. The arrow indicates the time of addition of IPTG (0.2 mM). Data are representative of at least three independent experiments and the error bars indicate the standard deviation. (**a**) For production of ferulic acid, a recombinant strain harboring *sam8-sam5-comt*/pACYC (red columns) or *tal-hpaBC-comt*/pACYC (dark cyan columns) was grown in Luria-Bertani (LB) medium with an additional 2 g/L l-tyrosine. Cell growth (lines; colors the same as those for ferulic acid production) is presented as the optical density at 600 nm. (**b**) Time course of vanillin production from ferulic acid in *E*. *coli* VT-3 cultures. (**c**) Production of vanillin from l-tyrosine using recombinant strains VT-4 and VT-5. (**d**) Fermentative production of vanillin from glucose. The control strain is *E. coli* strain K12 harboring *sam8*-*sam5*-comt/pACYC and *fcs*-*ech*/pET.

With the addition of the *comt* gene to the plasmids, the production of ferulic acid reached 156 mg/L (strain VT-1; *sam8*-*sam5*-*comt*/pACYC) and 43 mg/L (strain VT-2; *tal*-*hpaBC*-*comt*/pACYC) after 48 h of culture in the same medium and under the same conditions described above (Fig. 3a). The results indicated that ferulic acid production was more efficient with *sam8-sam5-comt*/pACYC than with *tal-hpaBC-comt*/pACYC, and plasmid *sam8-sam5-comt*/pACYC was therefore chosen for further research.

**Figure 4:**
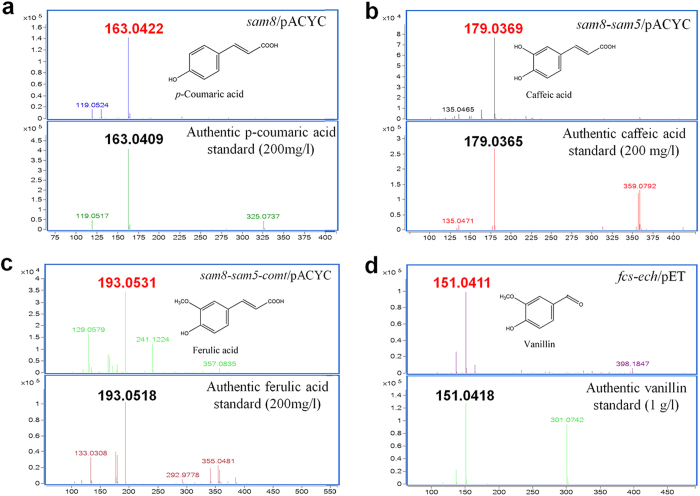
Mass spectrometry analysis of reaction products. Electrospray ionization mass spectra were obtained after liquid chromatography–mass spectrometry analysis of the culture medium of recombinant strains and of authentic standards; the corresponding mass spectra are shown in this figure. Negative ion data for the standard compounds were as follows: *p*-coumaric acid, m/z = 163.0401; caffeic acid, m/z = 179.0350; ferulic acid, m/z = 193.0506; resveratrol, m/z = 227.0714; naringenin, m/z = 271.0612; bisdemethoxycurcumin, m/z = 307.0976 and vanillin, m/z = 151.0415. (**a**) Sample from recombinant strain harboring *sam8*/pACYC (upper panel) and authentic 4-coumaric acid standard (lower panel). (**b**) Sample from recombinant strain harboring *sam8-sam5*/pACYC (upper panel) and authentic caffeic acid standard (lower panel). (**c**) Sample from recombinant strain harboring *sam8-sam5-comt*/pACYC (upper panel) and authentic ferulic acid standard (lower panel). (**d**) Sample from recombinant strain harboring *fcs-ech*/pET (upper panel) and authentic vanillin standard (lower panel).

The yields of 4-coumaric acid, caffeic acid, and ferulic acid were measured by HPLC. These products were also identified using LC–MS and compared with corresponding standards (Fig. 4). During all the experiments mentioned above, no 4-coumaric acid, caffeic acid, or ferulic acid was detected in the culture medium of wild-type *E. coli* carrying the control (blank) vector.

### Production of vanillin from tyrosine by recombinant *E. coli*

Vanillin production from recombinant *E. coli* harboring *fcs-ech*/pET (VT-3) was investigated using LB medium containing 1 g/L ferulic acid as substrate and 0.2 mM IPTG as inducer. In HPLC analysis, the retention time of the major product was identical to that of authentic vanillin; we further analyzed the compound by HPLC coupled to MS in the negative-ion mode and observed a molecular ion of *m/z* 151.04, indicating that this compound was indeed vanillin (Fig. 4). After 36 h of culture at 26 °C, the concentration of vanillin in the medium was 692 mg/L (Fig. 3b) and the corresponding molar conversion rate was 88.3%, which is high compared with previous studies^6^. This finding suggests that the strain harboring *fcs-ech*/pET can convert ferulic acid to vanillin with high efficiency and the plasmid is a very good choice for our artificial vanillin biosynthetic system.

**Figure 5:**
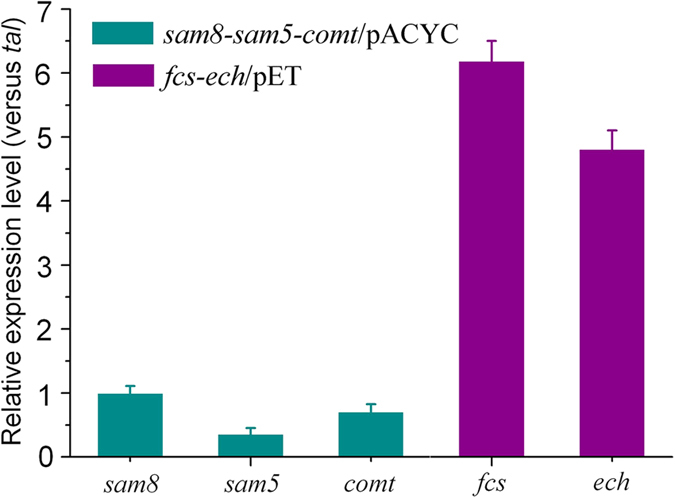
Relative transcription levels of vanillin biosynthetic pathway genes. Total RNA of recombinant strains VT-4 was extracted from glucose-based LB medium 8 h after induction with IPTG, and transcription levels were determined by quantitative RT-PCR. All values are relative to the expression level of the *sam8* gene, which was set at 1. Dark cyan and purple columns indicate the expression levels of the genes involved in the artificial pathways from tyrosine to ferulic acid and from ferulic acid to vanillin, respectively. Results are presented as the average of three repetitions from independent total RNA samples.

Based on these results, we combined the ferulic acid-producing pathway described above with the pathway for conversion of ferulic acid to vanillin. Thus, production of vanillin from tyrosine was carried out with strain VT-4 (recombinant *E. coli* harboring *sam8-sam5-comt*/pACYC and *fcs-ech*/pET). Strain VT-4 was grown in LB medium containing 2 g/L l-tyrosine and 0.2 mM IPTG, and 97.2 mg/L vanillin was obtained after 48 h of culture at 26 °C (Fig. 3c). The molar conversion of l-tyrosine to vanillin was 4.8%, which is relatively low. To determine the cause of the low conversion rate, we first examined the degradation of vanillin in the culture medium. Vanillin was added to a culture of strain VT-4 at a concentration close to the production yield. As shown in Supplementary Fig. 2, though vanillin was relatively stable in the culture medium, the degradation of a small amount of vanillin (less than 15% after 24 h) was one of the causes of the low molar conversion rate. To further investigate the low yield of vanillin from tyrosine, we examined the expression levels of the biosynthetic genes (*sam8*, *sam5*, *comt*, *fcs*, and *ech*) and the amounts of intermediate compounds. Quantitative reverse transcription PCR (RT-PCR) results indicated that all biosynthetic genes were expressed (Fig. 5). The genes in pACYCDuet-1 (*sam8*, *sam5*, and *comt*) had lower expression levels than the genes in pETDuet-1 (*fcs* and *ech*), and *sam8* and *comt* had higher expression levels than *sam5*. l-Tyrosine and intermediate compounds (caffeic acid and ferulic acid) were not detected in the culture medium, and about 29.2 mg/L 4-coumaric acid was detected. Besides, a small amount of vanillyl alcohol (about 35.7 mg/L) could be detected in the culture of recombinant strain VT-4. This is in accordance with the results that some aromatic aldehyde reductases in *E. coli* could convert vanillin to vanillyl alcohol^13^. Thus, the low conversion rate of tyrosine to vanillin may be due to the endogenous side-reactions and inefficient conversion of 4-coumaric acid to caffeic acid. Plasmid *fcs-ech-sam5*/pET, with an additional *sam5* gene, was constructed to replace plasmid *fcs-ech*/pET. As shown in Fig. S3, although RT-PCR results indicated that the expression level of *sam5* gene was improved in VT-5 (recombinant *E. coli* harboring *sam8-sam5-comt*/pACYC and *fcs-ech-sam5*/pET); the production of vanillin from VT-5 was similar to that from VT-4 (Fig. 3c). Furthermore, a small amount of 4-coumaric acid still remained in the culture of VT-4 (about 21.4 mg/L), indicating that the enzyme activity of C3H may be a critical factor for vanillin production in our system.

### Vanillin production from simple carbon sources

The recombinant *E. coli* strain containing the plasmid *tyrA*^*fbr*^*-aroG*^*fbr*^*-tktA-ppsA*/pCOLA and a knockout mutation in *tyrR* has been tested for its ability to produce tyrosine^27^. When 10 g/L glucose was used as a carbon source, the engineered *E. coli* strain produced l-tyrosine in LB medium at a titer of about 1.1 g/L after 24 h, and the corresponding molar conversion rate was 10.9%. In contrast, the wild-type *E. coli* strain produced low quantities of l-tyrosine.

The artificial vanillin biosynthetic pathway from l-tyrosine was constructed in the tyrosine-overproducing *E. coli* strain by transforming cells with two plasmids (*sam8-sam5-comt*/pACYC and *fcs-ech*/pET). Vanillin was produced from 10 g/L glucose at a titer of 19.3 mg/L, and the conversion rate was 0.193%. Without glucose, the yield of vanillin was below the detection limit. Moreover, the production of vanillin was quite low in the wild-type *E. coli* strain containing the biosynthetic pathway from tyrosine to vanillin (Fig. 3d). These results indicate that vanillin was derived from glucose and that metabolic engineering of the aromatic amino acid pathway was necessary for the production of vanillin from a simple carbon source.

**Figure 6:**
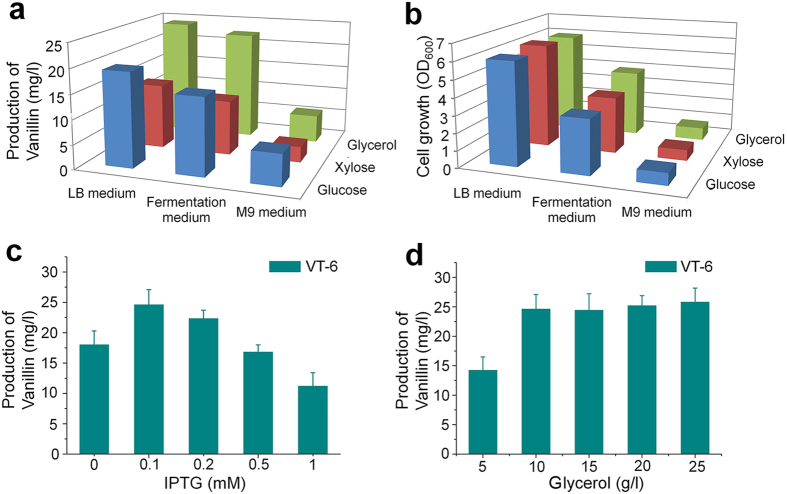
Optimization of vanillin production from simple carbon sources. Samples were collected 60 h after induction with IPTG and analyzed by HPLC. Data are representative of three independent experiments. (**a**) Production of vanillin by engineered strain VT-6 grown in different media with added glucose (blue column), xylose (brown column), or glycerol (green column). (**b**) Final cell density of engineered strain VT-6 grown in different media. (**c**) Effect of IPTG concentration on the production of vanillin. (**d**) Effect of glycerol concentration on the production of vanillin.

To reduce costs, additional experiments were performed with M9 minimal medium and fermentation medium supplemented with 10 g/L glucose. As shown in Fig. 6a, vanillin production of the recombinant *E. coli* strain in fermentation medium and M9 minimal medium was 15.7 mg/L and 6.4 mg/L, respectively. Compared with LB medium and fermentation medium, vanillin production was much lower in M9 minimal medium; this may have been due to the lower cell density (Fig. 6b), or the M9 minimal medium may have lacked metal cofactors needed for heterologous enzyme activity. Although vanillin production in fermentation medium was slightly lower than production in LB medium, the specific titer (vanillin titer divided by cell density) was roughly 1.5 times higher in fermentation medium (reaching 4.9 mg/L per OD unit) than in LB medium. This was probably the result of more of the carbon source in the LB medium consumed for cell growth.

Vanillin production from xylose and glycerol was also investigated. When 10 g/L xylose or 10 g/L glycerol was used as carbon source, the engineered *E. coli* strain produced vanillin in LB medium at a yield of 13.3 mg/L or 24.1 mg/L, respectively (Fig. 6a). Vanillin production was higher with glucose, a better carbon source for *E. coli* metabolism, than with xylose. On the other hand, the vanillin titer was lower with glucose than with glycerol. This is consistent with previous studies that found that glycerol is a more suitable carbon source than glucose for the production of shikimic acid and other compounds related to the shikimic acid pathway^27^,^31^. The recombinant *E. coli* strain was also cultivated in M9 minimal medium and fermentation medium with xylose or glycerol as substrate. As shown in Fig. 6a, the results were similar to those obtained when glucose was provided as the carbon source: the highest specific titer was achieved in fermentation medium and the lowest production was achieved in M9 minimal medium. These results indicate that glycerol is the best carbon source for vanillin production among the three substrates, and fermentation medium is the most cost-effective medium owing to the high specific titer and low cost.

To further optimize the culture conditions for the recombinant *E. coli* strain, glycerol was chosen as the substrate and fermentation medium was chosen as the preferred production medium. The highest vanillin production (24.7 mg/L) was observed when 0.1 mM IPTG was added to the medium (Fig. 6c). Higher concentrations of IPTG reduced the vanillin titer, and only 11.3 mg/L vanillin was detected in the medium when 1 mM IPTG was added; this may have been caused by a metabolic burden on the *E. coli* host. The “leaky” expression of biosynthetic genes without IPTG induction led to the production of vanillin at a yield of 18.1 mg/L; although a higher yield was achieved with 0.1 mM IPTG, the elimination of IPTG can reduce production costs. As shown in Fig. 6d, vanillin production in medium with 15 g/L, 20 g/L, or 25 g/L glycerol was 24.5 mg/L, 25.3 mg/L, and 25.9 mg/L, respectively; vanillin production in medium with 10 g/L glycerol was not significantly different. However, the production of vanillin decreased to 14.3 mg/L when 5 g/L glycerol was used as substrate. These results show that 10 g/L glycerol is sufficient for vanillin production.

## Discussion

Synthetic biology is the design and construction of biological devices and systems for useful purposes^32^. Demand for natural plant metabolites is increasing and synthetic biology has been widely used for the production of these compounds by microorganisms. However, the rational design of feasible pathways is one of the major challenges in this field, and artificial pathways have not always performed ideally in the host, often leading to low production^33^. Furthermore, an artificial pathway may use only one substrate, such as eugenol to vanillin by the biotransformation, which limits its application^5^. Simulating and assembling natural pathways in the host may counter these problems because natural pathways are the result of evolution over a long period and seem to be more efficient and stable. More importantly, natural synthetic pathways are often connected with basal metabolism^34^. This connection would enable the production of desired product from various available substrates, and the pathways can be easily transplanted to other production platforms. Many plant genes are quite challenging to functionally express in prokaryotic systems; therefore, for successful expression of mimicked natural pathways in microorganisms, microbial genes should be chosen instead of plant genes whenever possible. With the development of genomics and bioinformatics, more and more genes from microorganisms are becoming available for the construction of mimicked pathways.

In this study we succeeded in introducing a mimicked metabolic route for vanillin production into *E. coli*; with this recombinant strain, tyrosine, glucose, xylose, and glycerol can be used as substrate to produce vanillin. This is the first report of vanillin production from tyrosine by microbes and the first attempt to genetically engineer a single recombinant prokaryote for *de novo* biosynthesis of vanillin. Compared with previously developed artificial pathways for the production of vanillin from simple carbon sources^9^,^10^,^11^, the simulated natural pathway presented here has several advantages. One advantage is that the pathway can use l-tyrosine as substrate, which has less influence on the basal metabolism of the host; it is quite different from the previous artificial vanillin synthetic pathway that limited the biosynthesis of aromatic amino acids. Another advantage is that the simulated pathway can be transplanted to other tyrosine-overproducing strains to improve vanillin production^35^,^36^, and most of the genetic modification to improve the yield of tyrosine, such as co-expression of the rate-limiting enzymes shikimate kinase and quinate/shikimate dehydrogenase, also plays a role in increasing the yield of vanillin^37^. Moreover, expensive precursor chemicals or carbohydrate feedstocks are the main cost of microbial industry; with simulated natural pathways connected to primary metabolism, cheaper and more readily available carbon sources can be used for the production of desired compounds.

Although natural pathway mimicking has many advantages for the production of natural compounds, the yield of vanillin in our system was not very high. Analysis of the low conversion rate of tyrosine to vanillin indicated that it may have been due to the inefficient conversion of 4-coumaric acid to caffeic acid, endogenous side-reactions and, to a lesser extent, the instability of vanillin. In the future, the use of previous strategy to delete aldo-keto reductases and alcohol dehydrogenases may improve the conversion rate^13^. Furthermore, it will be necessary to look for more efficient enzymes that relate to this biosynthetic route and to C3H in particular. Because the artificial vanillin biosynthetic pathway will consume ATP and NADPH^21^,^26^, another strategy for improving the yield of vanillin is to balance NADP^+^ and ATP. There are various ways to achieve this; for example, to regenerate NADPH, which is the coenzyme of C3H, an NADPH-regenerating enzyme (glucose-6-phosphate dehydrogenase or phosphite dehydrogenase) should be introduced into the system^38^,^39^. Many methods of synthetic biology and fermentation technology, such as fed-batch fermentation or the use of bioreactors to increase cell density, may further boost the vanillin titer^16^. According to our recent research, choosing a suitable promoter is also an effective way to obtain high yields^40^.

To reduce production costs, we optimized the culture media, the concentration of IPTG, and the concentration of substrate. The results will guide the large-scale production of vanillin with our system. Besides glucose, xylose, and glycerol, many other inexpensive carbon sources, such as molasses, wheat flour, and rice bran, have been found to support microbial growth and enzyme production^41^. Combining a *de novo* vanillin biosynthetic pathway with these agro-industrial byproducts will offer a cost-effective process for vanillin production and may have remarkable economic benefits. Lignocellulosic biomass, the most abundant raw material on Earth, contains a lot of reducing sugars and aromatic compounds^42^. The products of its decomposition, including xylose, ferulic acid, and caffeic acid, can be used as substrates for vanillin production with our system. Here we have provided new perspectives and ideas for the simultaneous and effective utilization of complex components.

Recently, more and more plant metabolites were identified and proved with potential bioactivities, their metabolic pathways and key enzymes have also been clarified^41^. The rich diversity of microbes and explosive growth of microbial genome sequence data provide unprecedented gene resources that enable to mimick efficient pathways in microorganisms. Moreover, the rising demand of high-value plant metabolites will promote the use of this method. In conclusion, we have designed a simulated natural pathway to biosynthesize vanillin, making it possible to use microbes to produce vanillin from inexpensive carbon sources. This is a successful example of mimicking natural pathway for *de novo* biosynthesis by using cheap carbon sources for the efficient production of valuable plant metabolites.

## Methods

### Chemicals, bacterial strains and plasmids

All chemicals were purchased from Sigma-Aldrich (St. Louis, MO) unless otherwise specified. Restriction enzymes, ligase (New England Biolabs Inc.), and DNA polymerase (Takara Biochemicals Inc.) were used for cloning and plasmid construction. Oligonucleotides were synthesized by Sangon Biotech Co. (Shanghai, China). The characteristics of the bacterial strains and plasmids used in this study are provided in Table 2. *Saccharothrix espanaensis* (DSM 44229) and *R. sphaeroides* (DSM 158) were obtained from DSMZ. *E. coli* DH5α and *E. coli* BL21 (DE3) were used for general cloning and expression of biosynthetic genes in feeding experiments, respectively. The l-tyrosine- overproducing strain containing a knockout mutation in *tyrR* and a plasmid expressing the *aroG*^*fbr*^, *tyrA*^*fbr*^, *ppsA*, and *tktA* genes was a gift from Professor Minami and was used for shake flask experiments. A pEASY-Blunt cloning vector (Transgen, China) was used for subcloning of genes. Plasmids pACYCDuet-1 and pETDuet-1 were purchased from Novagen (San Diego, CA) and used for gene overexpression in *E. coli*.

### Bacterial cultivation conditions

*Streptomyces* sp. strain V-1 (CCTCC M 206065) was cultivated at 30 °C in seed medium, which contained 10 g/L glucose, 5 g/L yeast extract, 10 g/L peptone, 5 g/L beef extract, and 2 g/L NaCl (pH 7.0), as previously described^33^. *Escherichia coli* cells used for gene cloning, plasmid propagation, and inoculum preparation were cultured at 37 °C in LB medium supplemented with appropriate antibiotics. The working concentrations of antibiotics were 100 μg/mL for ampicillin, 50 μg/mL for kanamycin, and 20 μg/mL for chloromycetin. For production of 4-coumaric acid, caffeic acid, ferulic acid, and vanillin from tyrosine, 500 μL of overnight LB culture was inoculated into 50 mL of LB medium with 2 g/L l-tyrosine and grown at 37 °C. After the OD_600_ reached 0.5–0.6, IPTG was added to the cultures to a final concentration of 0.2 mM, and cultures were transferred to a gyratory shaker at 26 °C for 3 days. For *de novo* biosynthesis of vanillin, 500 μL of overnight LB culture was inoculated into 50 mL of LB, M9, or fermentation medium supplemented with 10 g/L glucose, xylose, or glycerol; the culture conditions were the same as those used for vanillin production from tyrosine. The fermentation medium was modified M9 minimal salt medium containing 1 g NH_4_Cl, 6 g Na_2_HPO_4_, 3 g KH_2_PO_4_, 0.5 g NaCl, 2 mmol MgSO_4_·7H_2_O, 0.1 mmol CaCl_2_·2H_2_O, and 0.5 g yeast extract per liter. Trace elements (0.03 mg/L H_3_BO_3_, 1 mg/L thiamine, 0.94 mg/L ZnCl_2_, 0.5 mg/L CoCl_2_, 0.38 mg/L CuCl_2_, 1.6 mg/L MnCl_2_, and 3.6 mg/L FeCl_2_) were added to the LB and fermentation media. Samples were collected at intervals of 6 or 12 h and analyzed by HPLC and LC–MS.

### Heterologous pathway construction and assembly

Genomic DNA was extracted from *R. sphaeroides*, *E. coli*, *S. espanaensis*, and *Streptomyces* sp. V-1 using a bacterial genomic DNA extraction kit (QIAGEN, Hilden, Germany). Lists of primers used in this study can be found in Tables S1 and S2. The genes *tal* (GenBank accession No. CP000144.2) and *sam8* (GenBank accession No. DQ357071) were amplified by high-fidelity PCR from the genomic DNA of *R. sphaeroides* and *S. espanaensis*, respectively. The resulting PCR products were cloned into the *Nco*I and *Eco*RI sites of pACYCDuet-1, resulting in *tal*/pACYC and *sam8*/pACYC. The genes *hpaBC* (GenBank accession No. CP001509) and *sam5* (GenBank accession No. DQ357071) were amplified from the genomic DNA of *E. coli* and *S. espanaensis*, respectively, and the *Nde*I–*Xho*I fragment of the resulting products was cloned into *tal*/pACYC and *sam8*/pACYC, generating *tal-hpaBC*/pACYC and *sam8-sam5*/pACYC, respectively. The *comt* gene from *A. thaliana* (GenBank accession No. NM124796) was codon-optimized, fused with a T7 promoter, and synthesized by recombinant PCR^43^; the primers used for recombinant PCR are listed in Table S2. After digestion with *Sac*I and *Not*I, *comt* was cloned into *tal-hpaBC*/pACYC and *sam8-sam5*/pACYC to generate *tal-hpaBC-comt*/pACYC and *sam8-sam5-comt*/pACYC. The genes *fcs* (GenBank accession No. KC847405) and *ech* (GenBank accession No. KC847406) were amplified by PCR from chromosomal DNA of *Streptomyces* sp. V-1. The *ech* and *fcs* genes were ligated into the *Nco*I and *Hin*dIII sites of Multiple Cloning Site (MCS) II and the *Nde*I and *Xho*I sites of MCS I of pETDuet-1, respectively, to construct *fcs-ech*/pET. With the primers HindIII-T7-F and NotI-*sam5*-R, the *sam5* gene with the T7 promoter was amplified from *sam8-sam5*/pACYC and cloned into *fcs-ech*/pET, generating *fcs-ech*-*sam5*/pET. Gene sequences and orientations were confirmed by nucleotide sequencing after each round of cloning. The maps for plasmids *sam8-sam5-comt*/pACYC and *fcs-ech*/pET are shown in Fig. 2. To construct the strains producing 4-coumaric acid, caffeic acid, and ferulic acid, *E. coli* BL21 (DE3) was transformed with *sam8*/pACYC, *sam8-sam5*/pACYC, and *sam8-sam5-comt*/pACYC, respectively. To construct the vanillin-producing strains, *sam8-sam5-comt*/pACYC and *fcs-ech*/pET (or *fcs-ech*-*sam5*/pET) were co-transformed into *E. coli* BL21 (DE3) or the l-tyrosine-overproducing strain.

### Quantitative RT-PCR analysis of synthetic pathway genes

The recombinant *E. coli* strain harboring *sam8-sam5-comt*/pACYC and *fcs-ech*/pET was grown in LB medium at 37 °C, and 0.2 mM IPTG was added to the culture when the OD_600_ reached 0.6. Cells were cultured on a gyratory shaker at 26 °C for 8 h and then collected for total RNA extraction using an RNAprep Pure Cell/Bacteria Kit (Tiangen Biotech Co., Beijing, China). RNA was quantified using a NanoVue spectrophotometer (GE Healthcare Bio-Sciences, Sweden). After removal of genomic and plasmid DNA from RNA preparations using DNase I (Thermo Scientific), a total of 2 μg of RNA was used in reverse transcription reactions with random primers and SuperScript III Reverse Transcriptase (Invitrogen, Shanghai, China). Meanwhile, the RNA sample was used as a temple, and the PCR reaction was performed to certify there is no plasmid DNA in the RNA sample. Relative RNA concentrations were determined by quantitative RT-PCR using a 7300 Real-time PCR system with RealMasterMix (SYBR Green) (Tiangen Biotech Co.). Primers were designed using Beacon Designer 8.12 and are listed in Table S1. The amount of mRNA was quantified against a standard curve using the C_T_ value.

### HPLC/ESI-MS analysis of cultures

Culture samples containing more than 500 mg/L l-tyrosine were alkalized to a final concentration of 0.25 M KOH and incubated for 30 min at room temperature to dissolve all l-tyrosine. All samples taken from the cultures were centrifuged at 15,000 × g for 10 min and the supernatants were filtered through a 0.2-μm syringe filter. The samples were analyzed by HPLC using an Agilent 1200 series instrument with an Eclipse XDB-C18 column (4.6 × 150 mm) and an Ultimate 3000 Photodiode Array Detector maintained at 25 °C. Vanillyl alcohol was analyzed according to the previous method^13^. Other compounds were analyzed use the following method. The flow rate was 1 mL/min and the mobile phase consisted of solvent A (0.1% trifluoroacetic acid in water) and solvent B (0.1% trifluoroacetic acid in acetonitrile). The following gradient elution program was used: 0 min, 95% solvent A + 5% solvent B; 8 min, 20% solvent A + 80% solvent B; 10 min, 80% solvent A + 20% solvent B; 11 min, 95% solvent A + 5% solvent B. Production of l-tyrosine was monitored by measuring the absorbance at 280 nm, and production of 4-coumaric acid, caffeic acid, ferulic acid, and vanillin was monitored by measuring the absorbance at 310 nm. The retention times of the five above-mentioned compounds were 3.4, 5.3, 4.7, 5.5, and 5.7 min, respectively. After HPLC, LC–MS was performed using an Agilent UPLC-TOF-MS system. Compounds were identified and quantified by comparing the observed retention times, peak areas, and mass chromatograms with those of the corresponding chemical standards. The data shown in this study were generated from at least three independent experiments and analyzed using Microsoft Office Excel 2007 and IBM SPSS Statistics.

## Additional Information

**How to cite this article**: Ni, J. *et al.* Mimicking a natural pathway for *de novo* biosynthesis: natural vanillin production from accessible carbon sources. *Sci. Rep.* **5**, 13670; doi: 10.1038/srep13670 (2015).

## Supplementary Material

Supplementary Information

## Figures and Tables

**Table 1:** Production of phenylpropanoic acids by recombinant *E. coli* strains.

*E. coli* BL21(DE3)	Product (mg/L)		
	4-Coumaricacid	Caffeicacid	Ferulicacid
*tal*/pACYC	821 ± 31^a^	—	—
*sam8*/pACYC	875 ± 46^a^	—	—
*tal-hpaBC*/pACYC	272 ± 17	44 ± 13^b^	—
*sam8-sam5*/pACYC	221 ± 34	136 ± 6^b^	—
*tal-hpaBC-comt*/pACYC	112 ± 21	41 ± 10	43 ± 9^c^
*sam8-sam5-comt*/pACYC	86 ± 11	37 ± 13	156 ± 12^c^

The recombinant *E. coli* strains were cultured in LB medium with 1.5 g/L l-tyrosine. The sign ‘—’ indicated that the compound not detected in HPLC analysis. The data are generated from at least triplicate independent experiments, and the standard deviations were shown. Different letter codes (a, b and c) indicate significant differences (a, P = 0.061; b, P = 0.00037; c, P = 0.0002).

**Table 2:** Plasmids and strains used in this study.

Plasmid or strain	Relevant characteristics	Source
Plasmid		
pEASY-Blunt	f1 ori, T7 promoter, Kan^R^ and Amp^R^	Transgen
pETDuet-1	f1 ori, T7 promoter, double T7 promoter, Amp^R^	Novagen
pACYCDuet-1	p15A ori, double T7 promoter, Cm^R^	Novagen
*tyrA*^*fbr*^*-aroG*^*fbr*^*-tktA-ppsA*/pCOLA	pCOLADuet-1 contained the *tyrA*^*fbr*^, *aroG*^*fbr*^, *tktA*, and *ppsA* genes	[25]
*tal*/pACYC	pACYCDuet-1 contained the *tal* gene	This study
*sam8*/pACYC	pACYCDuet-1 contained the *sam8* gene	This study
*tal-hpaBC*/pACYC	pACYCDuet-1 contained the *tal* and *hpaBC* genes	This study
*sam8-sam5*/pACYC	pACYCDuet-1 contained the *sam8* and *sam5* genes	This study
*tal-hpaBC-comt*/pACYC	pACYCDuet-1 contained the *tal*, *hpaBC* and *comt* genes	This study
*sam8-sam5-comt*/pACYC	pACYCDuet-1 contained the *sam8*, *sam5* and *comt* genes	This study
*fcs-ech*/pET	pETDuet-1 contained the *fcs* and *ech* genes	This study
*fcs-ech-sam5*/pET	pETDuet-1 contained the *fcs, ech* and *sam5*genes	This study
Strain		
*E. coli* DH5a	Cloning host	Invitrogen
*E. coli* BL21(DE3)	Expression host	Novagen
*S. espanaensis* DSM 44229	Used for cloning the *sam8* and *sam5* genes	From DSMZ
*R. sphaeroides* DSM 158	Used for cloning the *tal* gene	From DSMZ
Tyrosine overproducing strain	ΔtyrR harboring pCOLA-TATP	[25]
VT-1	*E. coli* BL21(DE3) harboring *sam8-sam5-comt*/pACYC	This study
VT-2	*E. coli* BL21(DE3) harboring *tal-hpaBC-comt*/pACYC	This study
VT-3	*E. coli* BL21(DE3) harboring *fcs-ech*/pET	This study
VT-4	*E. coli* BL21(DE3) harboring *sam8-sam5-comt*/pACYC and *fcs-ech*/pET	This study
VT-5	*E. coli* BL21(DE3) harboring *sam8-sam5-comt*/pACYC and *fcs-ech-sam5*/pET	This study
VT-6	Tyrosine overproducing strain harboring *sam8-sam5-comt*/pACYC and *fcs-ech*/pET	This study
