# Supplementary material for: Mimicking a natural pathway for de novo biosynthesis: natural vanillin production from accessible carbon sources
Source: Sci Rep. 2015 Sep 2;5:13670. doi: 10.1038/srep13670 (PMC4557066; doi:10.1038/srep13670)
Supplement: Supplementary Information [file srep13670-s1.pdf]

Supplementary Information

Mimicking a natural pathway for *de novo* biosynthesis: natural vanillin production from accessible carbon sources

Jun Ni, Fei Tao, Huaiqing Du and Ping Xu[§]

State Key Laboratory of Microbial Metabolism, and School of Life Sciences & Biotechnology, Shanghai Jiao Tong University, Shanghai 200240, People's Republic of China.

[§]Correspondence: Ping Xu

Mailing address: School of Life Sciences and Biotechnology, Shanghai Jiao Tong University, Shanghai 200240, People's Republic of China

E-mail: pingxu@sjtu.edu.cn (P. Xu)

Tel: 86-21-34206647; Fax: 86-21-34206723

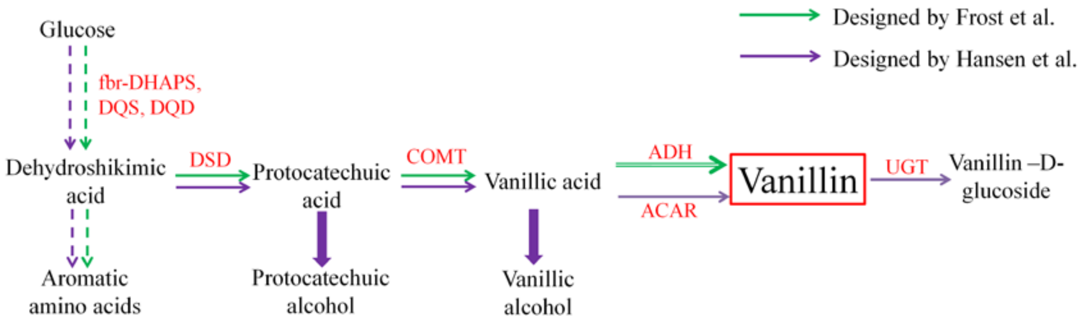

Figure S1. Biosynthetic scheme for *de novo* biosynthesis of vanillin in previous researches. Green lines indicates the pathway designed by Frost et al. Purple lines indicates the pathway designed by Hansen et al. Dotted lines indicates the common biosynthetic pathway of aromatic amino acid; double line indicates the *in vitro* step; broad lines indicates the undesired metabolic reactions catalyzed by the host alcohol dehydrogenases. fbr-DAHPS, fbr-3-deoxy-d-arabino-heptulosonate-7-phosphate synthase; DQS, dehydroquinate synthase; DQD, dehydroquinate dehydratase; DSD; dehydroshikimate dehydratase; COMT, catechol-O-methyltransferase; ADH, aryl-aldehyde dehydrogenase; ACAR, aromatic carboxylic acid reductase; UGT, UDP-glycosyltransferase.

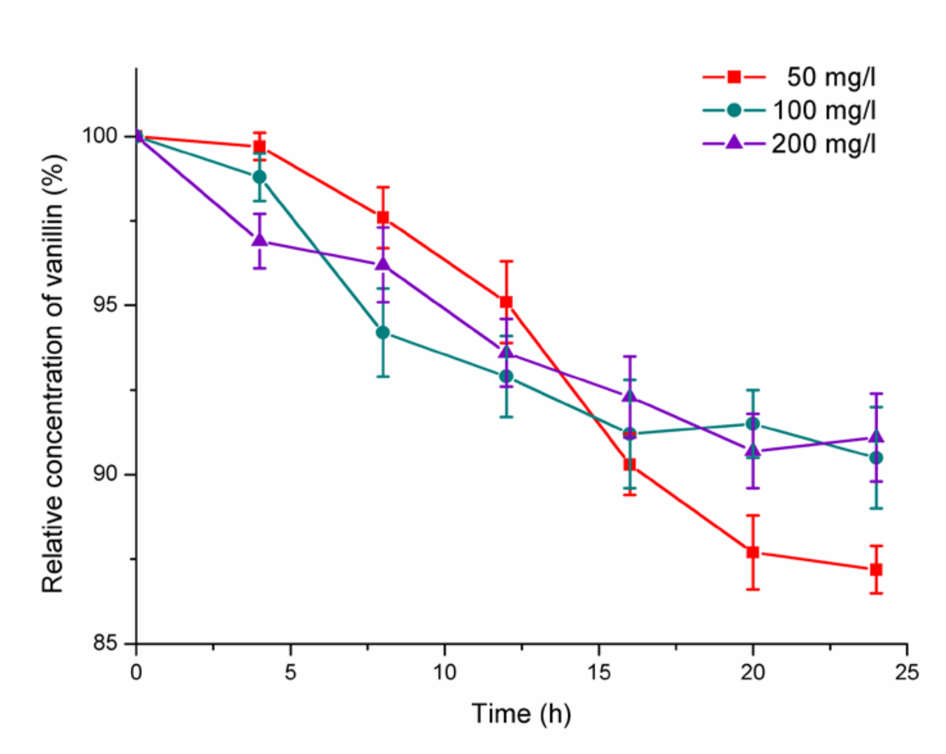

Figure S2. Degradation of vanillin in the culture medium. Vanillin was added to the LB culture of recombinant strain VT-4 at a final concentration of 50 mg/l (red squares), 100 mg/l (green circles) and 200 mg/l (purple triangles), the remaining amount of vanillin was compared with the additive amount. Before addition, the strain was treated with 0.2 mM IPTG for 18 h to the stationary phase.

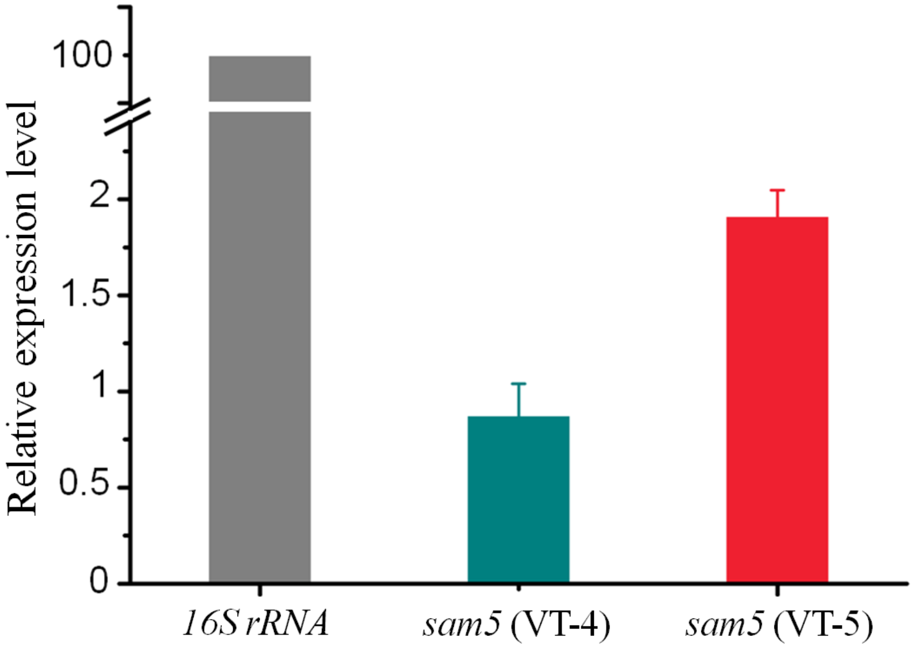

Figure S3. Relative transcription levels of 4-coumarate 3-hydroxylase gene. All values are relative to the expression levels of corresponding 16S rRNA gene, which were set at 100. Green and red columns indicate the expression levels of the *sam5* in recombinant strains VT-4 and VT-5, respectively. Results are presented as the average of six repetitions (triplicate reverse transcription reactions from two independent total RNA samples).

Table S1. DNA sequences of the primers used in this study.

Primer	Sequence (5' to 3')
NcoI-*sam8*-F [a]	CATGCCATGGGCATGACGCAGGTCGTGGAACG
EcoRI-*sam8*-R [a]	CATGGAATTCTTATCCGAAATCCTTCCCGT
sam8-F[b]	GAGTACCTGAAGTCCGACAAG
sam8-R[b]	CTGCTCCAGCTCCGAGTC
NcoI-*tal*-F [a]	CATGCCATGGGCATGCTCGCCATGAGCCCC
EcoRI-*tal*-R [a]	CATGGAATTCTTAGACGGGAGATTGCTGCAAG
NdeI-*sam5*-F [a]	CATGCATATGACCATCACGTCACCTGC
XhoI-*sam5*-R [a]	CATGCTCGAGTTAGGTGCCGGGGTTGATCA
HindIII-T7-F [a]	CATGAAGCTTTTGTACACGGCCGCATAATC
NotI-*sam5*-R [a]	CATGGCGGCCGCTTAGGTGCCGGGGTTGATCA
sam5-F[b]	GGTGCGGGACAAGAAGTTC
sam5-R[b]	TAGGAGGTGCGGCAGATG
NdeI-*hpaBC*-F [a]	CATGCATATGATGAAACCAGAAGATTTCCGCGC
XhoI-*hpaBC*-R [a]	CATGCTCGAGTTAAATCGCAGCTTCCATTTCCAGC
EcoRI-T7-F [a]	CATGGAGCTCCGAAATTAATACGACTCACTATAGGGGAATTGTGAGC
NotI-*comt*-R [a]	CATGGCGGCCGCTTACAGTTTTTTCAGCAGT
comt-F[b]	GCTATCCTGGACGGTGGTATC
comt-R[b]	TGGTGATGGTAGAGTGGTTAGAC
NdeI-*fcs*-F [a]	CATGGAATTCCATATGCGCAACCAGGGTCTGGGC
XhoI-*fcs*-R [a]	CATGCTCGAGTCAGCCGAAGCGGCGGCGGACCTCGCC
fcs-F[b]	GTGTGCCTGCTGATGTAC
fcs-R[b]	CAGTTCCAGGTGAGGTTG
NcoI-*ech*-F [a]	CATGCCATGGGCATGAGCACAGCGGTCGGCAACGGG
HindIII-*ech*-R [a]	CATGAAGCTTCTACTTCTCCGGGTCGAAGGCGCTCAG
ech-F[b]	ACGGTTCTGGTGGAGTTC
ech-R[b]	ACCATCTCGTCGTTCAGG
16S rRNA-F[b]	ACGAGTGGCGGACGGGTG
16S rRNA-R[b]	CACATCCGATGGCAAGAGGC

[a] Underlining indicates restriction enzyme cleavage sites corresponding to the primer description.

[b] The DNAs are used for quantitative RT-PCR.

Table S2. DNA sequences of primers used for the synthesis of *comt* gene (codon-optimized and fused with T7 promoter).

Primer	Sequence (5' to 3')
1 [a] (EcoRI-T7-F)	CATGGAGCTCCGAAATTAATACGACTCACTATAGGGGAATTGTGAGC
2	TATTTCTAGAGGGGAATTGTTATCCGCTCACAATTCCCCTATAGTGA
3	GGATAACAATTCCCCTCTAGAAATAATTTTGTTTAACTTTAAGAAGGAGATATAC
4	TTTCAGCGGTAGAACCCATGGTATATCTCCTTCTTAAAGTTAAACAAAAT
5	CATGGGTTCTACCGCTGAAACCCAGCTGACCCCGGTTCAGGTTAC
6	TAGCGAACAGAGCAGCTTCGTCGTCGGTAACCTGAACCGGGGTCA
7	GAAGCTGCTCTGTTCGCTATGCAGCTGGCTTCTGCTTCTGTTCTG
8	CCAGAGCAGATTTCAGAGCCATCGGCAGAACAGAAGCAGAAGCCA
9	GGCTCTGAAATCTGCTCTGGAACTGGACCTGCTGGAAATCATGGC
10	TCGGAGACATCGGAGAACCGTTTTTAGCCATGATTTCCAGCAGGT
11	GGTTCTCCGATGTCTCCGACCGAAATCGCTTCTAAACTGCCGACC
12	AGCATAACCGGAGCTTCCGGGTTTTTGGTCGGCAGTTTAGAAGCG
13	GGAAGCTCCGGTTATGCTGGACCGTATCCTGCGTCTGCTGACCTC
14	CGGTTAGAGCAGGTCAGAACAGAGTAAGAGGTCAGCAGACGCAGG
15	GTTCTGACCTGCTCTAACCGTAAACTGTCTGGTGACGGTGTTGAA
16	CAAACCGGACCCAGACCGTAGATACGTTCAACACCGTCACCAGAC
17	CGGTCTGGGTCCGGTTTGCAAATACCTGACCAAAAACGAAGACGG
18	CAGGCACAGAGCAGCGATAGAAACACCGTCTTCGTTTTTGGTCAG
19	TCGCTGCTCTGTGCCTGATGAACCAGGACAAAGTTCTGATGGAAT
20	AGCGTCTTTCAGGTGGTACCAAGATTCCATCAGAACTTTGTCCTG
21	GTACCACCTGAAAGACGCTATCCTGGACGGTGGTATCCCGTTCAA
22	CGAAAGCAGACATACCGTAAGCTTTGTTGAACGGGATACCACCGT
23	CTTACGGTATGTCTGCTTTCGAATACCACGGTACCGACCCGCGTT
24	AGACATACCGTTGTTGAAAACTTTGTTGAAACGCGGGTCGGTACC
25	AAGTTTTCAACAACGGTATGTCTAACCACTCTACCATCACCATGA
26	CTTTGTAGGTTTCCAGGATTTTTTTCATGGTGATGGTAGAGTGGT
27	AAAAAATCCTGGAAACCTACAAAGGTTTCGAAGGTCTGACCTCTC
28	CGATACCACCACCAACGTCAACCAGAGAGGTCAGACCTTCGAAAC
29	GACGTTGGTGGTGGTATCGGTGCTACCCTGAAAATGATCGTTTCT
30	ACCTTTCAGGTTCGGGTATTTAGAAACGATCATTTTCAGGGTAG
31	AAATACCCGAACCTGAAAGGTATCAACTTCGACCTGCCGCACGTT
32	ATACCCGGGTGAGACGGAGCGTCTTCGATAACGTGCGGCAGGTCG
33	CCGTCTCACCCGGGTATCGAACACGTTGGTGGTGACATGTTCGTT
34	AGATAGCGTCACCTTTCGGAACAGAAACGAACATGTCACCACCAA
35	TCCGAAAGGTGACGCTATCTTCATGAAATGGATCTGCCACGACTG
36	GAATTTAACGCAGTGTTCGTCAGACCAGTCGTGGCAGATCCATTT
37	TGACGAACACTGCGTTAAATTCCTGAAAAACTGCTACGAATCTCT
38	GATAACTTTACCGTCTTCCGGCAGAGATTCGTAGCAGTTTTTCAG
39	GCCGGAAGACGGTAAAGTTATCCTGGCTGAATGCATCCTGCCGGA

40	TTGGTAGACAGAGAAGAGTCCGGGGTTTCCGGCAGGATGCATTCA
41	CGGACTCTTCTCTGTCTACCAAACAGGTTGTTCACGTTGACTGCA
42	CCACCCGGGTTGTGAGCCAGCATGATGCAGTCAACGTGAACAACC
43	CTCACAACCCGGGTGGTAAAGAACGTACCGAAAAAGAATTCGAAG
44	AAACCAGAAGCTTTAGCCAGAGCTTCGAATTCTTTTTCGGTACGT
45	TCTGGCTAAAGCTTCTGGTTTCAAAGGTATCAAAGTTGTTTGCGA
46	GATCAGGTTAACACCGAAAGCGTCGCAAACAACTTTGATACCTTT
47	GCTTTCGGTGTTAACCTGATCGAACTGCTGAAAAAACTGTAAGCG
48[a] (NotI-*comt*-R)	CATGGCGGCCGCTTACAGTTTTTTCAGCAGT

[a] Underlining indicates restriction enzyme cleavage sites corresponding to the primer description.
